# High-density microprojection array delivery to rat skin of low doses of trivalent inactivated poliovirus vaccine elicits potent neutralising antibody responses

**DOI:** 10.1038/s41598-017-13011-0

**Published:** 2017-10-03

**Authors:** David A. Muller, Germain J. P. Fernando, Nick S. Owens, Christiana Agyei-Yeboah, Jonathan C. J. Wei, Alexandra C. I. Depelsenaire, Angus Forster, Paul Fahey, William C. Weldon, M. Steven Oberste, Paul R. Young, Mark A. F. Kendall

**Affiliations:** 1 Delivery of Drugs and Genes Group (D2G2) Australian Institute for Bioengineering and Nanotechnology, The University of Queensland, Brisbane, Queensland QLD 4072 Australia; 2 Australian Infectious Diseases Research Centre, The University of Queensland, Brisbane, Queensland Australia; 3 ARC Centre of Excellence in Convergent Bio-Nano Science and Technology, Queensland, Australia; 4 School of Chemistry and Molecular Biosciences, The University of Queensland, Brisbane, Queensland Australia; 5 Vaxxas Pty Ltd, Translational Research Institute, Brisbane, Queensland 4102 Australia; 6 Division of Viral Diseases, National Center for Immunization and Respiratory Diseases, Centers for Disease Control and Prevention, Atlanta, Georgia USA

## Abstract

To secure a polio-free world, the live attenuated oral poliovirus vaccine (OPV) will eventually need to be replaced with inactivated poliovirus vaccines (IPV). However, current IPV delivery is less suitable for campaign use than OPV, and more expensive. We are progressing a microarray patch delivery platform, the Nanopatch, as an easy-to-use device to administer vaccines, including IPV. The Nanopatch contains an ultra-high density array (10,000/cm^2^) of short (~230 μm) microprojections that delivers dry coated vaccine into the skin. Here, we compare the relative immunogenicity of Nanopatch immunisation versus intramuscular injection in rats, using monovalent and trivalent formulations of IPV. Nanopatch delivery elicits faster antibody response kinetics, with high titres of neutralising antibody after just one (IPV2) or two (IPV1 and IPV3) immunisations, while IM injection requires two (IPV2) or three (IPV1 and IPV3) immunisations to induce similar responses. Seroconversion to each poliovirus type was seen in 100% of rats that received ~1/40th of a human dose of IPV delivered by Nanopatch, but not in rats given ~1/8th or ~1/40th dose by IM injection. Ease of administration coupled with dose reduction observed in this study suggests the Nanopatch could facilitate inexpensive IPV vaccination in campaign settings.

## Introduction

When the global poliovirus eradication effort began in 1988, poliovirus was endemic in over 125 countries causing >350,000 cases of paralysis each year. Through extensive use of the attenuated oral poliovirus vaccine (OPV), polioviruses are on the verge of eradication – with only 37 cases of wild type poliovirus 1 reported in 2016, and with only 2 countries being endemic for poliovirus, Pakistan and Afghanistan^1^. The last case of wild type poliovirus 2 was in 1999, with the virus officially declared eradicated in September 2015^2^ and type 3 transmission has been interrupted since 2012^3^ – leaving type 1 as the only wild poliovirus still circulating. While the oral vaccine is very safe, a small number of individuals (1 in 2.7 million vaccinees^4^) can suffer from vaccine-associated poliovirus paralysis (VAPP). Furthermore, after several rounds of replication in the gut, OPV can revert to a neurovirulent phenotype, which can be spread to naïve individuals in the community through faecal contamination. These events can result in the circulation of vaccine-derived polioviruses (cVDPV), which were responsible for 32 cases of poliovirus vaccine associated disease in 2015^1^. Although wild poliovirus type 2 has been eradicated, vaccine-derived poliovirus 2 has been responsible for the majority of cVDPV and sporadic VAPP cases^1,5^. Therefore, in order to achieve a world free of circulating polioviruses, the live attenuated OPV will need to be replaced with the inactivated poliovirus vaccine (IPV). As the first stage in this transition occurred in April 2016 where there was a synchronised global shift from trivalent OPV to bivalent OPV (bOPV types 1 and 3). With type 2 immunity now being provided by at least one injection of IPV^6^ incorporated into all routine immunization schedules. This shift has put further demand on an already constrained IPV supply^7^.

To date, a large part of the success of the poliovirus eradication campaign has been due to the relative ease with which the vaccine can be administered in mass campaigns. The introduction of IPV poses several challenges to the eradication effort, not least because door-to-door campaigns will become more logistically difficult. Needle and syringe administration of IM injections will necessitate a campaign based on the efforts of healthcare professionals with more training than that needed for administration of OPV drops. In addition, as IPV is introduced to mass vaccination campaigns, the cost of the vaccine will be a major factor, with a single dose of IPV costing more than five times that of OPV^8,9^. As a result of these factors, there are several investigations are underway to make IPV more accessible, by reducing the cost of vaccination, through stretching the already constrained supply of IPV through dose-sparing and by increasing ease of administration^10,11^. These dose sparing approaches include: use of adjuvants^12^, ID injectors^13–16^, jet injectors^17–22^ and finally microarray patch (MAP) technologies^10,11^. The use of ID injection using standard ID needle and syringes is also being clinically investigated^13^ for poliovirus vaccination. These fractional dose approaches have a two-fold benefit: the cost per dose should decrease and more doses should become available. One form of MAP is the Nanopatch: designed to directly deliver vaccine to the skin’s abundant immune cell populations with a high density array, to improve vaccine potency and thereby open up significant dose sparing, compared to the needle and syringe^23^. Demonstrated clinical dose sparing, embodied in a practical, low-cost device could allow the Nanopatch to help improve vaccination campaigns in low resource regions^24^, including poliovirus eradication.

**Figure 1:**
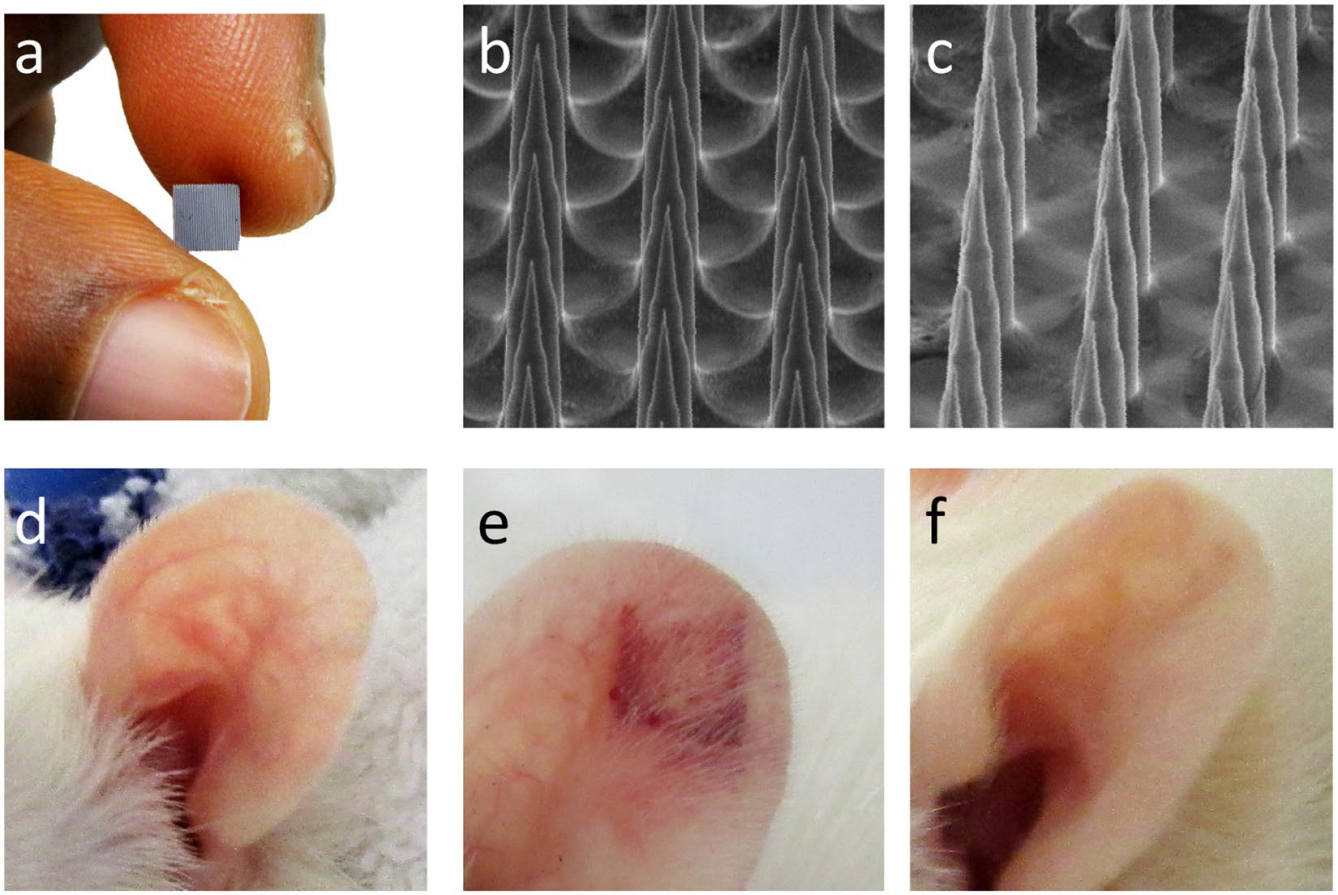
Nanopatch coating and application (**a**) 4 mm^2^ Nanopatch (**b**) representative image of IPV coated Nanopatch (**c**) SEM image of Nanopatch following application. Representative images of a Wistar rat ear (**d**) before, (**e**) immediately after and (**f**) 1 week post application.

Here we examine a microneedle skin patch, the Nanopatch, as a viable alternative to the traditional needle and syringe to deliver IPV. The Nanopatch (prototype used in rats) is a 4 × 4 mm silicon microarray patch (Fig. 1a), containing 10,000 microprojections per cm^2^ at 230 μm in length to which vaccine is dry coated for delivery into the skin. The vaccine-coated Nanopatch is applied dynamically using a spring-loaded applicator to the skin^25^. This allows the projections to penetrate through the stratum corneum, depositing vaccine to antigen-presenting cells in both the viable epidermis and dermis. Previous studies have shown that localised cell death and inflammation associated with the dynamic application, coupled with vaccine delivery, results in improved immune responses as compared to traditional needle and syringe approaches^26^. In exploring the utility of this mode of action, delivery of multi-valent vaccines by Nanopatches to mice has generated improved immune responses and dose sparing to each strain of split, trivalent influenza vaccines^23,24,27^, virus-like particle HPV vaccine^28^, 9-valent subunit conjugated vaccine (pneumococcal)^29^, DNA vaccines (West Nile virus)^30^ and attenuated live-virus vaccines (adenovirus and MVA)^31^.

Turning to polio, recently we applied Nanopatches to demonstrate the induction of potent neutralising antibodies directed against poliovirus type 2 using monovalent IPV2 vaccine with as little as 0.2 DU (1/40^th^ of a standard human dose) in the Wistar rat model^11^. However, the question of whether these benefits extend to trivalent poliovirus vaccine in the rat IPV immunogenicity model remains unanswered.

In this study, we extend our monovalent IPV2 studies to Nanopatch delivery of all three monovalent IPVs as well as a single Nanopatch application with trivalent IPV in Wistar rats. We show that using the Nanopatch enhances the anti-poliovirus antibody responses to all 3 types of poliovirus over that induced by intramuscular injection by needle and syringe.

## Results

### Nanopatch coating and application

Based on our previous studies^11^, we established IPV formulations containing either each IPV type individually, or all three poliovirus serotypes in a single formulation. We evaluated Nanopatch coating and application by SEM of coated Nanopatches before (Fig. 1b) and after application (Fig. 1c), to ensure consistent coating morphology and removal of coated vaccine after application. Nanopatch applications were performed as described below, with no serious adverse events noted. Immediately following application (n = 10), there was reddening and some petechiae with the greatest effect observed at the edges of the patched area (Fig. 1e). This is consistent with previous work^26,32^, both reporting greater rates of cell death at the edges of Nanopatch application sites generated by higher stresses^33^. During the course of one week, all observable local skin reactions following Nanopatch application were resolved (Fig. 1f), with the ear returning to a pre-vaccination appearance (as in Fig. 1d).

### Triple monovalent IPV immunogenicity study

We performed a dose-matched immunogenicity study where rats received 3 immunisations with IPV spaced 21 days apart. On each occasion, three Nanopatches were applied (one serotype per patch) to each animal or the three monovalent formulations were given by separate intramuscular injections. We chose doses based on previous experience with monovalent IPV2 for which 1 and 0.2 D-antigen units (DU) of IPV2 were delivered, and extrapolated the IPV1 and 3 doses using the same ratios as used in the full human IPV doses. Therefore we delivered 5 DU of IPV1, 1 DU of IPV2 and 4 DU of IPV3, which corresponds to 1/8^th^ of a human dose. We also tested a low-dose of 1 DU IPV1, 0.2 DU of IPV2 and 0.8 DU of IPV3 (1/40^th^ of a human dose). As a positive control, one group of rats received a full human dose (40:8:32 DU of types 1, 2, and 3, respectively) by IM injection. To confirm the desired dose of each IPV was deposited into the ear of the rats we performed D-antigen ELISAs on the coated formulation remaining on the Nanopatch post application. Results are presented in Figure s1a–b demonstrate our ability to deliver approximately 1/8^th^ (IPV1: 5.9 ± 0.6 DU, IPV2: 1.3 ± 0.25 DU, IPV3: 5.2 ± 0.3 DU) and 1/40^th^ (IPV1: 1.3 ± 0.16 DU, IPV2: 0.17 ± 0.03 DU, IPV3: 0.89 ± 0.1) of a full human IPV dose by Nanopatch. Blood samples were collected prior to each vaccination for evaluation of poliovirus neutralising antibody titres.

**Figure 2:**
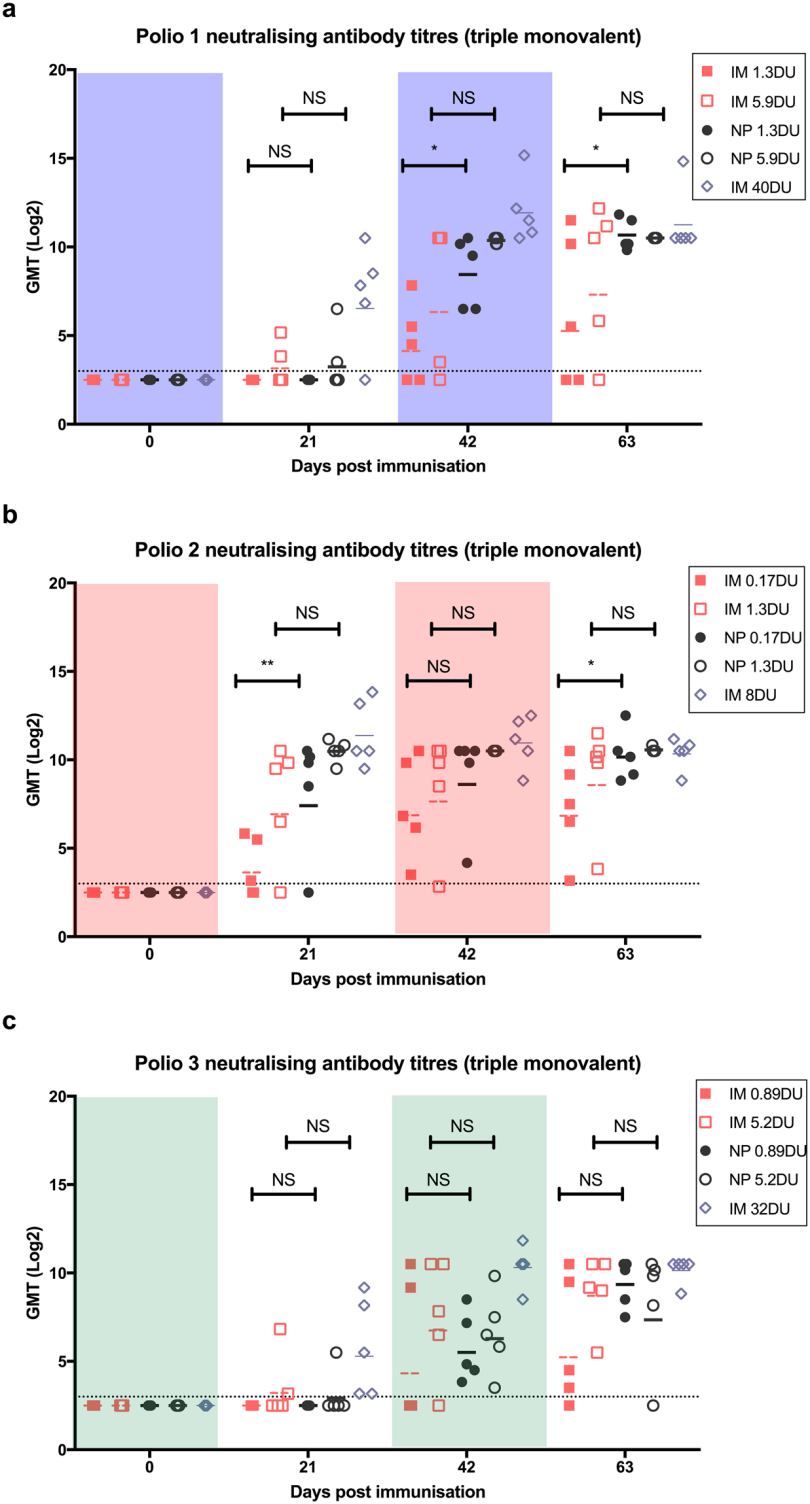
Poliovirus neutralising antibody responses to (**a**) IPV1, (**b**) IPV2 and **(c)** IPV3 following triple monovalent vaccination. Vaccine doses used were a standard human dose 40:8:32 DU, DU, for types 1, 2, and 3, respectively), ~1/8^th^ of the human dose (5.9:1.3:5.2 DU) and 1/40^th^ of the human dose (1.3:0.17:0.89 DU). A positive response is defined as a neutralising titre ≥3.0 log_2_ (dotted line). Each symbol represents a single animal, with the line indicating the median titre. * and ** indicates a statistically significant difference between dose matched Nanopatch and IM group as assessed by one-way ANOVA (alpha level 0.05) with a Sidak post test of p = <0.05 and p = <0.01, respectively.

All rats were negative for neutralising antibody (log_2_ titre < 3) before commencement of the study. Following the first vaccination, 40% of animals receiving 5.9 DU of IPV1 seroconverted (log_2_ titre ≥ 3) with relatively low titres; no rats receiving 1.3 DU of IPV1 seroconverted (Figure s2a). However, following the first boost, 100% of animals receiving IPV1 by Nanopatch seroconverted, whereas 60% and 80% seroconversion was observed for the 1.3 and 5.9 DU IM groups, respectively. As compared to their IM counterparts, the Nanopatch groups produced higher antibody titres, with 1.3 DU Nanopatch producing a significantly higher (p < 0.05) titre of anti-poliovirus 1 antibody (Fig. 2a). Following the second boost, all median titres increased again with the Nanopatch groups producing more anti-poliovirus 1 antibody as compared to IM injection. Median titres continued to rise with each boost, with 1.3 DU by Nanopatch producing significantly higher titres than its IM counterpart. For 5.9 DU the Nanopatch appeared to induce higher titres of neutralizing antibody compared to IM injection, albeit not statistically significant. Overall, Nanopatch delivery of IPV1 was superior to IM immunization with all animals seroconverting with high titres.

The antibody responses to monovalent IPV2 had similar kinetics and titres to results we previously reported^11^, suggesting that co-administration of the other IPV types on different Nanopatches did not result in either positive or negative immunological interference. Following the first dose, 100% and 80% of animals receiving 1.3 DU or 0.17 DU, respectively, by Nanopatch seroconverted, whilst 60% and 80% seroconverted in the equivalent IM groups (Figure s1b). Consistent with the IPV1 results, the animals receiving vaccine by Nanopatch had higher poliovirus neutralising antibody titres throughout the entire study (Fig. 2b), with each subsequent boost raising anti-poliovirus titres. All of Nanopatch animals seroconverted after the 2^nd^ dose.

IPV3 produced the weakest immune response regardless of delivery method. Following the first vaccination only 20% and 40% of animals receiving 5.2 DU by Nanopatch or IM, respectively, seroconverted (Figure s2c). No animals receiving 0.89 DU seroconverted after the first dose. After the first boost, 100% of animals receiving the vaccine via the Nanopatch seroconverted while only 40% and 80% seroconverted following IM immunization for the 0.89 or 5.2 DU respectively. After the third dose, all titres continued to increase; however, there was no significant difference between the titres induced by IM or Nanopatch delivery at either of the dose levels (Fig. 2c). Intriguingly, one of the rats receiving Nanopatch 5.2 DU titre declined below the limit of detection to IPV3 while maintaining relatively high titres against IPV 1 and 2.

### Trivalent IPV immunogenicity study

With ease of application in mind, we then endeavoured to formulate all 3 types of IPV onto a single Nanopatch, to deliver either 5:1:4 or 1:0.2:0.8 DU of IPVs 1, 2 and 3, respectively; i.e. 1/8th and 1/40th of the standard human dose. In order to coat the required doses of antigen onto a single 4 × 4 mm Nanopatch, we concentrated the monovalent bulk vaccine stocks. The 10x bulk vaccine was formulated with a cellulose carrier and stabilizing sugars to final concentrations of 1% methylcellulose, 0.75% trehalose, 0.75% sucrose and 0.35% mannitol.

**Figure 3:**
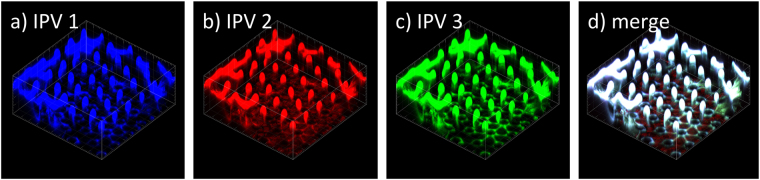
Representative images of fluorescently labelled tracer poliovirus to demonstrate homogenous composition of the 3 IPV types coated onto the Nanopatch. The coating solution contained 1% methyl cellulose, 0.75% trehalose, 0.75% sucrose, 0.35% mannitol and **(a)** IPV1 with dylight 647 shown in blue, (**b**) IPV2 with dylight 550 shown in red, (**c**) IPV3 with dylight 488 shown in green. (**d**) merge of images a-c with areas of complete overlap shown as white. Images representative of n = 4 replicates.

To confirm uniform distribution of each vaccine type within the coating formulation, we labelled live-attenuated poliovirus types 1, 2 and 3 with dylight 647, 550 and 488, respectively, and introduced them to the concentrated IPV as a tracer. Upon coating, each of the 3 types of poliovirus was visualised uniformly coated onto the projections (representative images seen in Fig. 3a–d). Following conformation we had developed a uniform coating process with consistent dose delivery for both the 1/8^th^ (IPV1: 4.7 ± 0.6 DU, IPV2: 0.8 ± 0.1 DU, IPV3: 4.1 ± 1 DU) and 1/40^th^ (IPV1: 1.7 ± 0.25 DU, IPV2: 0.26 ± 0.06, IPV3: 0.7 ± 0.06 DU) Nanopatch groups (Figure s1c–d), we proceeded to immunize Wistar rats using the same doses and vaccination schedule as above.

**Figure 4:**
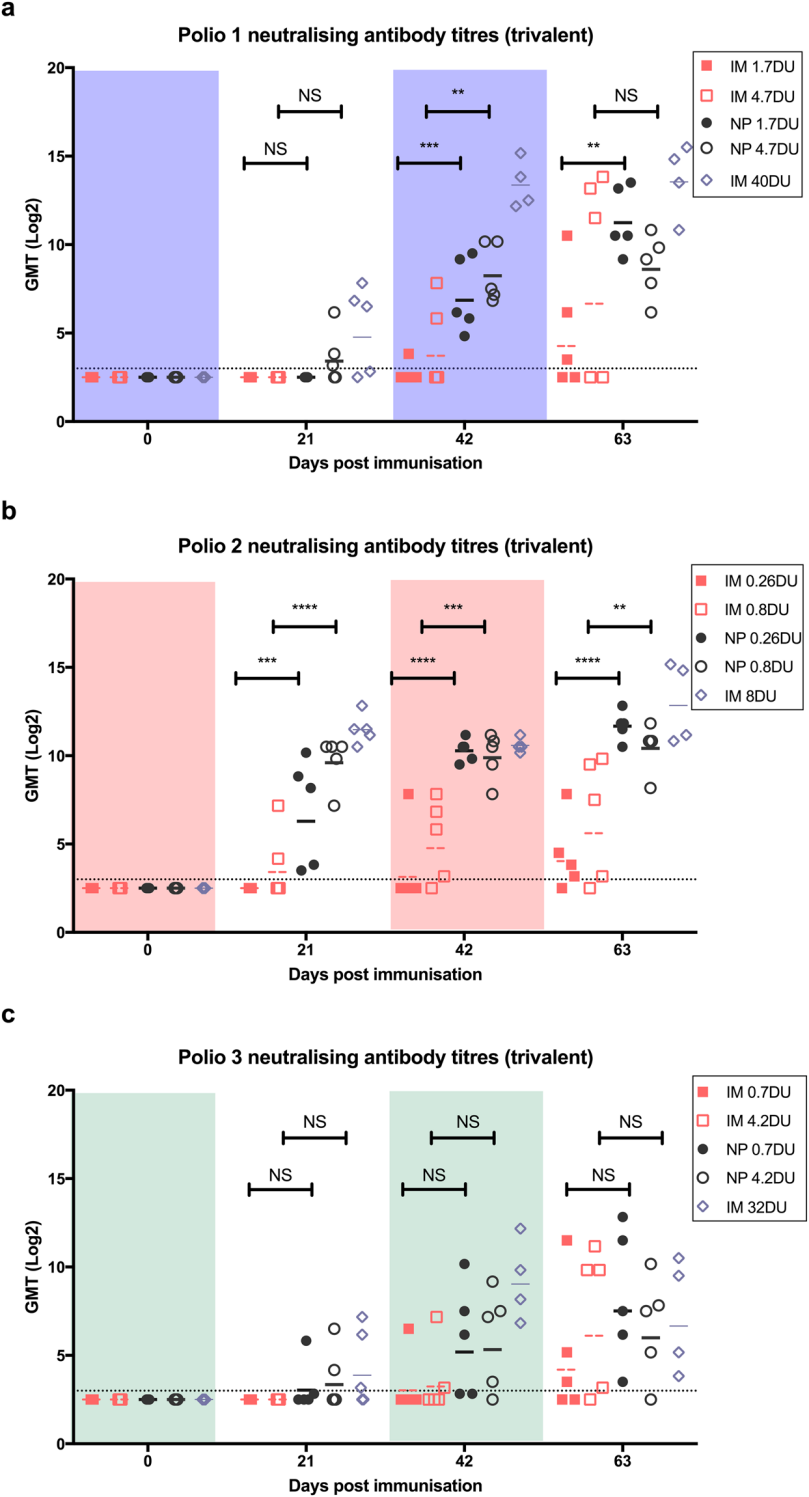
Poliovirus neutralising antibody responses to (**a**) IPV1, (**b**) IPV2 and (**c**) IPV3 following trivalent vaccination. Vaccine doses used were a standard human dose (types 1, 2, and 3–40:8:32 DU), 1/8^th^ of the human dose (4.7:0.8:4.3 DU) and 1/40^th^ of the human dose (1.7:0.26:0.7 DU). A positive response is defined as a neutralising titre ≥3.0 log_2_ (dotted line). Each symbol represents a single animal, with the line indicating the median titre. *, **, *** and **** indicates a statistically significant difference between dose matched Nanopatch and IM group as assessed by one-way ANOVA (alpha level 0.05) with a Sidak post test of p = <0.05, p = <0.01, p = <0.001 and p = <0.0001, respectively.

After each vaccination, blood was collected and analysed for the presence of poliovirus neutralising antibody. For IPV1, following the first dose of vaccine delivered by the Nanopatch, protective levels of neutralizing antibody were seen in 60% of animals while no neutralization was seen in serum of animals receiving the vaccine by IM. Upon boosting, all animals receiving vaccine via the Nanopatch had significantly higher (determined by one-way ANOVA, alpha level 0.05, with a Sidak post test) poliovirus neutralising titres than their dose-matched IM counterparts (Fig. 4a), while seroconversion rates of 20 and 40% were found for the low and high IM doses, respectively (Figure s3a). Following the second boost, the median neutralizing titre of each group continued to rise, with the low-dose Nanopatch producing significantly (determined by one-way ANOVA, alpha level 0.05, with a Sidak post test) higher titres than the dose-matched IM group. Notably, even after three immunizations by the IM route, not all animals produced neutralising poliovirus 1 antibodies (40% and 60% for the low- and high-dose, respectively). By contrast, after the 3^rd^ dose of Nanopatch-delivered vaccine, even the low-dose group showed high median neutralizing antibody titres comparable to, if not slightly higher than that of the high-dose Nanopatch group, a trend that was observed in the responses to all three poliovirus types.

All animals vaccinated by Nanopatch seroconverted to type 2 (Figure s3b) after a single dose (both 1/40^th^ and 1/8^th^ doses), producing significantly higher antibody titres than IM vaccination (determined by one-way ANOVA, alpha level 0.05, with a Sidak post test). With each subsequent boost, the median titres of all the groups continued to rise with the Nanopatch groups producing significantly higher neutralising antibody (determined by one-way ANOVA, alpha level 0.5, with a Sidak post test) responses than the dose-matched IM groups (Fig. 4b). Overall, the findings for IPV2, whether delivered in a monovalent, triple monovalent or trivalent formulation via the Nanopatch, showed the induction of potent neutralising antibodies from just a single dose.

Following the first dose, 20 and 40% of animals receiving the low and high dose of vaccine by Nanopatch (Figure s3c) seroconverted to poliovirus type 3. Type 3 neutralising antibody titres and seroconversion rates increased with each subsequent dose of vaccine (Fig. 4c). Following the final immunisation, 80% seroconversion was seen for the high dose of both Nanopatch and IM groups, while 100% and 60% seroconversion was achieved for the low-dose Nanopatch and IM groups, respectively. Consistent with the triple monovalent study, animals immunised with the Nanopatch tended to have higher antibody titres when compared to IM immunisation. Immunisation with both IPV1 and IPV2 elicit similar poliovirus neutralising titres and seroconversion rates when formulated in a single monovalent or trivalent IPV Nanopatch, but the monovalent IPV3 formulation resulted in higher overall median titres than when formulated in a trivalent IPV Nanopatch. However, similar rates of seroconversion were observed with both formulations.

## Discussion

In this study we have demonstrated that IPV of all three poliovirus serotypes can be formulated onto a single MAP device, the Nanopatch, and elicit neutralising antibody responses to all three poliovirus strains in rats. Furthermore, we demonstrated that there is no difference in response if IPV is delivered in either a monovalent or trivalent formulation, suggesting no immune competition. The fractional doses (1/8^th^ and 1/40^th^) used in this study when delivered by Nanopatch gave similar rates of seroconversion and median poliovirus neutralising titres to a full human dose delivered by IM injection, and after fewer doses. If the results from these studies translate from the rat model to humans, this could significantly reduce the dose required to produce an effective neutralising antibody response.

Several studies by other groups seeking to evaluate dose sparing by skin injection using alternative delivery methods have been carried out. It has been demonstrated in a pre-clinical and clinical setting that ID vaccination using 1/5^th^ of a dose can elicit similar responses as a full human dose delivered by IM injection^13–15^ and is now being used in India and Sri Lanka. Here we set out to improve upon our previous IPV2 monovalent^11^ studies by extending Nanopatch immunization to all 3 poliovirus serotypes. We delivered 2 fractional doses of IPV via the Nanopatch, at 1/8^th^ or 1/40^th^ of a standard IPV dose, 40:8:32 DU of poliovirus 1, 2, and 3, respectively. These doses were delivered in two formats, either with one IPV type per patch (therefore 3 patches/immunisation) or all 3 IPV types formulated on to a single trivalent Nanopatch. Regardless of Nanopatch format, anti-poliovirus antibody kinetics and median titres were similar or better than IM immunisation at each measured time point. Reponses to IPV2 were similar to our previously published data, with bridging IPV2 studies in which 1 DU was given by either one or two Nanopatches demonstrating similar kinetics and titres as previous experiments^11^. While the same seroconversion rates and dose-sparing effects were not achieved for types 1 and 3 as with IPV2, animals vaccinated by the Nanopatch still had overall higher titres, faster anti-poliovirus antibody kinetics, and higher seroconversion rates than their IM counterparts. In addition, the relative immunogenicity of the three IPV types was the same regardless of whether they were delivered by Nanopatch or IM injection. Furthermore, after the full vaccination course, animals receiving a full human dose IM or by Nanopatch had no significant difference in terms of antibody titre or seroconversion rate. Within the triple monovalent and trivalent experiments the Nanopatch groups receiving a 1/8^th^ fractional dose showed a faster initial induction of anti-IPV antibody responses in comparison to the 1/40^th^ fractional dose. At the completion of both studies, however, animals achieved similar titres against all IPV types whether receiving the 1/8^th^ or 1/40^th^ fractional doses by Nanopatch. We hypothesize that once an initial immune response is established against IPV the Nanopatch may provide a booster effect to the pre-existing immune response - possibly through a physical immune enhancer effect within the skin’s immune environment by its dynamic application - resulting in an overall similar IPV titre being achieved by either fractional dose group. In support of this Soonawala, *et al*. demonstrated that a 1/5^th^ fractional IPV dose delivered I.D. by Pharmajet inject was effective at boosting pre-existing polio immunity^21^. The studies presented here are the first microarray patch studies to demonstrate significantly higher antibody titres for poliovirus 1 and 2 from a single trivalent IPV formulation. While IPV3 did not elicit a significantly higher antibody response when delivered by Nanopatch in comparison to IM, there was still significant dose sparing, with 1/40^th^ dose of IPV3 producing similar titres to the full human IPV3 dose. While the lack of immune enhancement observed in the IPV3 responses is somewhat unexpected given the higher immune responses elicited with the Nanopatch for IPV1 and 2, there is precedent for this result. Previously, it has been reported that IPV3 produces relatively low responses compared to IPV1 and 2, when using fractional doses and/or solid IPV formulations^10,34,35^. This suggests the IPV3 component of the vaccine is possibly (1) less immunogenic than IPV1 and 2, or (2) not in excess. Combined these results suggest there maybe a need to optimise the ratio of each IPV component for delivery by the Nanopatch to achieve maximal virus neutralising titres in vaccine recipients.

The introduction of IPV as a supplement to OPV in the routine vaccination schedule has presented several challenges that the Nanopatch is designed to help address. Firstly, with utility in low resource settings in mind^22^, the Nanopatch is relatively easy to administer and conceptually can be delivered by minimally trained individuals, such as community health volunteers, as OPV is delivered now. Furthermore, while not the focus of this paper, with new developments in coating methods we have observed enhanced delivery efficiencies beyond 85%^36^. Secondly, by uniquely targeting the rich environment of immune cells in the skin (e.g. as discussed in^13,14^), the Nanopatch potentially is only required to deliver a fractional dose of IPV to elicit a comparable rate of seroconversion and anti-poliovirus titres as observed for the traditional needle and syringe immunisations by the IM route. Effectively translating the results here in rats to humans could thus significantly reduce the cost of vaccination with IPV, increase the availability of the vaccine and facilitate its administration in door-to-door campaigns.

## Materials and Methods

### Inactivated poliovirus vaccine formulation

Monovalent bulk inactivated poliovirus vaccine types 1 (1,553 DU/mL), 2 (763 DU/mL) and 3 (912 DU/mL) were supplied by Bilthoven Biologicals, Bilthoven, the Netherlands. In order to coat the required dose of IPV in an appropriately small volume, the IPV bulks were concentrated 10-fold by using a Centricon (Millipore) 100 kDa filter at 2,500 × g at 4 °C. Final vaccine concentration was determined by D-antigen ELISA to be 15,500 DU/mL, 7,600 DU/mL and 9,100 DU/mL for types 1–3 respectively.

### Nanopatch fabrication and coating

Nanopatch microprojection arrays were produced at a 230 μm, 10,000 projections/cm^2^ density as previously described by Jenkins *et al*.^37^ at the Melbourne Centre of Nanofabrication. The IPV stocks were mixed into a solution with the final concentration of 1% methylcellulose, 0.75% sucrose, 0.75% trehalose and 0.35% mannitol under a nitrogen gas jet stream, adapting methodologies described^38,20^.

### Determination of delivered dose

To determine the vaccine delivered by the Nanopatch into the ear of the rat we performed the D-antigen ELISA. The amount of vaccine delivered was determined by applying the Nanopatch to the rat ear then eluting the remaining vaccine off the patch in 50 μl of PBS and comparing that sample to coated unapplied Nanopatches. The D-antigen ELISA was performed as previously described by Edens^10^ with the following changes: IPV3 antibody HYB 300-05-02(Thermo Fisher Scientific) and the assay was developed as described by Muller *et al*.^11^.

### Wistar rats

Female 6–8 week old Wistar rats were purchased from the Animal Resource Centre, Perth and housed in the AIBN animal facility. All methods performed in this study were carried out in accordance with National Health & Medical Research Council guidelines and approved by The University of Queensland Animal Ethics Committee.

### Rat immunisation studies

Immunisation studies were based on previously published IPV studies^11^. Wistar rats (n = 5 in each group) were given 3 vaccinations 21 days apart by either Nanopatch or IM injection, with blood samples collected (tail tip bleed) one day before each vaccination and 21 days after the final dose. For the triple monovalent IPV study, rats received fractional doses of 5:1:4 (1/8^th^) or 1:0.2:4 DU of poliovirus types 1–3, respectively, under Ketamine and xylazine sedation. An additional positive control group was included that received a full human dose of 40:8:32 DU by IM injection. Three Nanopatches (one for each serotype) were applied for 2 minutes to the ventral ear pinnae using an applicator at a velocity of 3.1 m/s. IM injections were administered to the hind leg. For the trivalent IPV study, the vaccine doses and groups were kept the same as the triple monovalent study except the Nanopatches were formulated with all types of IPV delivered by a single Nanopatch.

### Poliovirus neutralisation assay

To evaluate the immune responses to each of the 3 polioviruses following vaccination virus neutralising titres were determined at the CDC in Atlanta, GA, USA. Neutralisation assays were performed as described by Weldon *et al*.^39^.

### Fluorescent labelling of poliovirus

The labeling of vaccine was done using Dylight Microscale antibody labeling kit (Thermo Fisher Scientific, the buffers and columns, resin from the 550 kit (Catalogue # 84531)) and 50 μg vials of Dylight amine-reactive dyes of each colour used according to the manufacturer’s instructions. Briefly, vaccines were prepared by dialyzing 3 ml of each IPV vaccine was against 5 L PBS at 4 °C overnight and for 2–4 hours against 2 L PBS the next day in Slide-A-Lyzer G2 Dialysis cassettes (20 K MWCO, Catalogue #87735, Thermo Fisher Scientific), then buffer exchanged with 0.05 M borate buffer prepared from the provided 0.67 M stock using Amicon Ultra-4 ml (Ultracel 30K®) centrifugal filter units (Merck-Millipore, Catalogue #UFC803024) at 4000 × g for 5 min, five times.

Prepared vaccine (100 μL) was added to the vial of Dylight reagent, vortexed and mixed by gently pipetting up and down. Reaction was then incubated for 1 hour at room temperature in the dark. The reaction mixture was purified for labeled vaccine using provided columns pre-calibrated with purification resin spun at 1000 × g, with labeled vaccine collected in tubes and stored at 4 °C protected from light prior to imaging.

### Nanopatch imaging

Coated Nanopatches were visualised on a Zeiss 510 confocal microscope, using a 20 × 0.8 NA air objective (Zeiss). Dylight 647, 550 and 488 signals were acquired at 5 µm intervals with 1.28 µs pixel/dwell and 12-bit depth. All three channels were collected sequentially, using the following settings: red 633 helium neon laser with a HFT 458/543/633 splitter and filter BP 650–710; blue 488 argon laser with a HFT 488/KP700 and filter LP505; green 543 helium neon laser with a HFT 488/543 and meta detector set to 553–692 nm. Image analyses including 3D reconstruction of the images were performed with Imaris software x64 6.3.1 (Bitplane AG, Zurich, Switzerland).

### Statistical analysis

Graphpad Prism 7.0a for Mac. Multiple comparison analysis was performed using one-way ANOVA with the alpha level set at 0.05 and a Sidak post test to compare dose-matched groups.

### Data availability

The datasets generated during and/or analysed during the current study are available from the corresponding author on reasonable request.

## Electronic supplementary material

Supplementary information

## References

[1] Morales M, Tangermann RH, Wassilak SG (2016). Progress Toward Polio Eradication - Worldwide, 2015–2016. MMWR. Morbidity and mortality weekly report.

[2] Toole MJ (2016). So close: remaining challenges to eradicating polio. BMC medicine.

[3] Kew OM (2014). Possible eradication of wild poliovirus type 3–worldwide, 2012. MMWR. Morbidity and mortality weekly report.

[4] WHO. *Vaccine-associated paralytic polio (VAPP) and vaccine-derived poliovirus (VDPV*, http://www.who.int/immunization/diseases/poliomyelitis/endgame_objective2/oral_polio_vaccine/VAPPandcVDPVFactSheet-Feb2015.pdf (2015).

[5] Hagan JE (2015). Progress toward polio eradication - worldwide, 2014–2015. MMWR. Morbidity and mortality weekly report.

[6] Duintjer Tebbens RJ, Hampton LM, Thompson KM (2016). Implementation of coordinated global serotype 2 oral poliovirus vaccine cessation: risks of potential non-synchronous cessation. BMC infectious diseases.

[7] UNICEF. *Inactivated Polio Vaccine: Supply Update*, http://www.unicef.org/supply/files/IPV_supply_update_May_2015.pdf (2015).

[8] UNICEF. *OPV*, http://www.unicef.org/supply/files/OPV. pdf (2015).

[9] UNICEF. *Inactivated Polio vaccine*, http://www.unicef.org/supply/files/IPV.pdf (2014).

[10] Edens C, Dybdahl-Sissoko NC, Weldon WC, Oberste MS, Prausnitz MR (2015). Inactivated polio vaccination using a microneedle patch is immunogenic in the rhesus macaque. Vaccine.

[11] Muller DA (2016). Inactivated poliovirus type 2 vaccine delivered to rat skin via high density microprojection array elicits potent neutralising antibody responses. Scientific reports.

[12] Andreasen LV, Hansen LB, Andersen P, Agger EM, Dietrich J (2015). Aluminium hydroxide potentiates a protective Th1 biased immune response against polio virus that allows for dose sparing in mice and rats. Vaccine.

[13] Cadorna-Carlos J, Vidor E, Bonnet MC (2012). Randomized controlled study of fractional doses of inactivated poliovirus vaccine administered intradermally with a needle in the Philippines. International journal of infectious diseases: IJID: official publication of the International Society for Infectious Diseases.

[14] Anand A (2015). Early priming with inactivated poliovirus vaccine (IPV) and intradermal fractional dose IPV administered by a microneedle device: A randomized controlled trial. Vaccine.

[15] Troy SB (2015). Comparison of the Immunogenicity of Various Booster Doses of Inactivated Polio Vaccine Delivered Intradermally Versus Intramuscularly to HIV-Infected Adults. J Infect Dis.

[16] Nelson KS, Janssen JM, Troy SB, Maldonado Y (2012). Intradermal fractional dose inactivated polio vaccine: a review of the literature. Vaccine.

[17] Resik S (2015). Immune responses after fractional doses of inactivated poliovirus vaccine using newly developed intradermal jet injectors: a randomized controlled trial in Cuba. Vaccine.

[18] Resik S (2015). Needle-free jet injector intradermal delivery of fractional dose inactivated poliovirus vaccine: Association between injection quality and immunogenicity. Vaccine.

[19] Resik S (2010). Randomized controlled clinical trial of fractional doses of inactivated poliovirus vaccine administered intradermally by needle-free device in Cuba. J Infect Dis.

[20] Resik S (2013). Priming after a fractional dose of inactivated poliovirus vaccine. N Engl J Med.

[21] Soonawala D (2013). Intradermal fractional booster dose of inactivated poliomyelitis vaccine with a jet injector in healthy adults. Vaccine.

[22] Clarke E (2016). Safety and immunogenicity of inactivated poliovirus vaccine when given with measles-rubella combined vaccine and yellow fever vaccine and when given via different administration routes: a phase 4, randomised, non-inferiority trial in The Gambia. The Lancet. Global health.

[23] Fernando GJ (2010). Potent immunity to low doses of influenza vaccine by probabilistic guided micro-targeted skin delivery in a mouse model. PLoS One.

[24] Chen X (2011). Improving the reach of vaccines to low-resource regions, with a needle-free vaccine delivery device and long-term thermostabilization. J Control Release.

[25] Crichton ML (2010). The effect of strain rate on the precision of penetration of short densely-packed microprojection array patches coated with vaccine. Biomaterials.

[26] Depelsenaire AC (2014). Colocalization of cell death with antigen deposition in skin enhances vaccine immunogenicity. The Journal of investigative dermatology.

[27] Chen X (2012). Rapid kinetics to peak serum antibodies is achieved following influenza vaccination by dry-coated densely packed microprojections to skin. J Control Release.

[28] Corbett HJ, Fernando GJ, Chen X, Frazer IH, Kendall MA (2010). Skin vaccination against cervical cancer associated human papillomavirus with a novel micro-projection array in a mouse model. PLoS One.

[29] Pearson FE (2015). Functional anti-polysaccharide IgG titres induced by unadjuvanted pneumococcal-conjugate vaccine when delivered by microprojection-based skin patch. Vaccine.

[30] Prow TW (2010). Nanopatch-targeted skin vaccination against West Nile Virus and Chikungunya virus in mice. Small.

[31] Pearson FE (2013). Dry-coated live viral vector vaccines delivered by nanopatch microprojections retain long-term thermostability and induce transgene-specific T cell responses in mice. PLoS One.

[32] Coffey JW, Meliga SC, Corrie SR, Kendall MA (2016). Dynamic application of microprojection arrays to skin induces circulating protein extravasation for enhanced biomarker capture and detection. Biomaterials.

[33] Meliga, S. C. *et al*. The Hyperelastic and Failure Behaviors of Skin in Relation to the Dynamic Application of Microscopic Penetrators in a Murine Model. *Acta biomaterialia*, 10.1016/j.actbio.2016.10.021 (2016). 10.1016/j.actbio.2016.10.021 27746361

[34] Kraan H (2015). Alternative delivery of a thermostable inactivated polio vaccine. Vaccine.

[35] Tzeng SY (2016). Thermostabilization of inactivated polio vaccine in PLGA-based microspheres for pulsatile release. J Control Release.

[36] Chen X (2011). Site-Selectively Coated, Densely-Packed Microprojection Array Patches for Targeted Delivery of Vaccines to Skin. Adv Funct Mater.

[37] Jenkins D, Corrie SR, Flaim C, Kendall MA (2012). High density and high aspect ratio solid micro-nanoprojection arrays for targeted skin vaccine delivery and specific antibody extraction. RSC Advances.

[38] Chen X (2009). Dry-coated microprojection array patches for targeted delivery of immunotherapeutics to the skin. J Control Release.

[39] Weldon WC, Oberste MS, Pallansch MA (2016). Standardized Methods for Detection of Poliovirus Antibodies. Poliovirus: Methods and Protocols.

