# Supplementary material for: High-density microprojection array delivery to rat skin of low doses of trivalent inactivated poliovirus vaccine elicits potent neutralising antibody responses
Source: Sci Rep. 2017 Oct 3;7:12644. doi: 10.1038/s41598-017-13011-0 (PMC5626768; doi:10.1038/s41598-017-13011-0)
Supplement: Supplementary file 1 — Supplementary information [file 41598_2017_13011_MOESM1_ESM.pdf]

Title: **High-density microprojection array delivery to rat skin of low doses of trivalent inactivated poliovirus vaccine elicits potent neutralising antibody responses.**

Authors Names and affiliations:

Authors Names and affiliations:

David A. Muller[1,2,4*], Germain J.P. Fernando[1,3], Nick S. Owens[1], Christiana Agyei-Yeboah[1], Jonathan C. J. Wei[1], Alexandra C.I. Depelsenaire[1], Angus Forster[5], Paul Fahey[5], William C. Weldon[6], M. Steven Oberste[6], Paul R. Young[2,4], Mark A. F. Kendall[1,2,3*].

[1]Delivery of Drugs and Genes Group (D2G2) Australian Institute for Bioengineering and Nanotechnology, The University of Queensland, Brisbane, Queensland QLD 4072, Australia.

[2]Australian Infectious Diseases Research Centre, The University of Queensland, Brisbane, Queensland, Australia.

[3]ARC Centre of Excellence in Convergent Bio-Nano Science and Technology, Queensland, Australia

[4]School of Chemistry and Molecular Biosciences, The University of Queensland, Brisbane, Queensland, Australia.

[5]Vaxxas Pty Ltd, Translational Research Institute, Brisbane, Queensland 4102, Australia.

[6]Division of Viral Diseases, National Center for Immunization and Respiratory Diseases, Centers for Disease Control and Prevention, Atlanta, Georgia, USA.

*To Whom Correspondence can be addressed:

Dr David A Muller

Address: The University of Queensland, Australian Institute for Bioengineering and Nanotechnology, Bulding 75, St Lucia, Queensland, 4072, Australia.

Email: d.muller4@uq.edu.au Tel: +61 7 3365 4881 Fax: +61 7 3346 4197

Supplementary information:

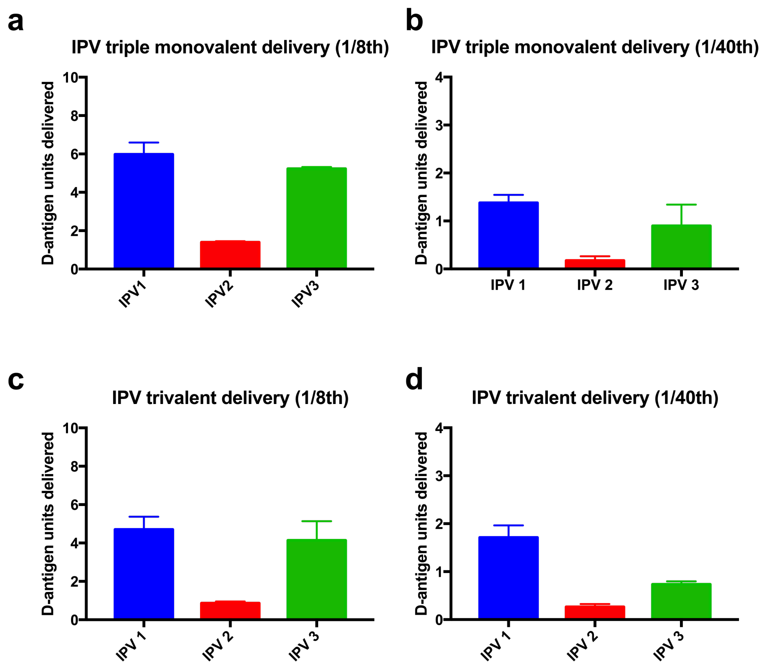

Figure S1: IPV delivered by Nanopatch into the ear of wistar rats as determined by D-antigen ELISA. Triple monovalent Nanopatch delivery of (**a**) 1/8th or (**b**) 1/40th IPV fraction doses. Trivalent Nanopatch delivery of (**c**) 1/8th or (**d**) 1/40th IPV fraction doses.

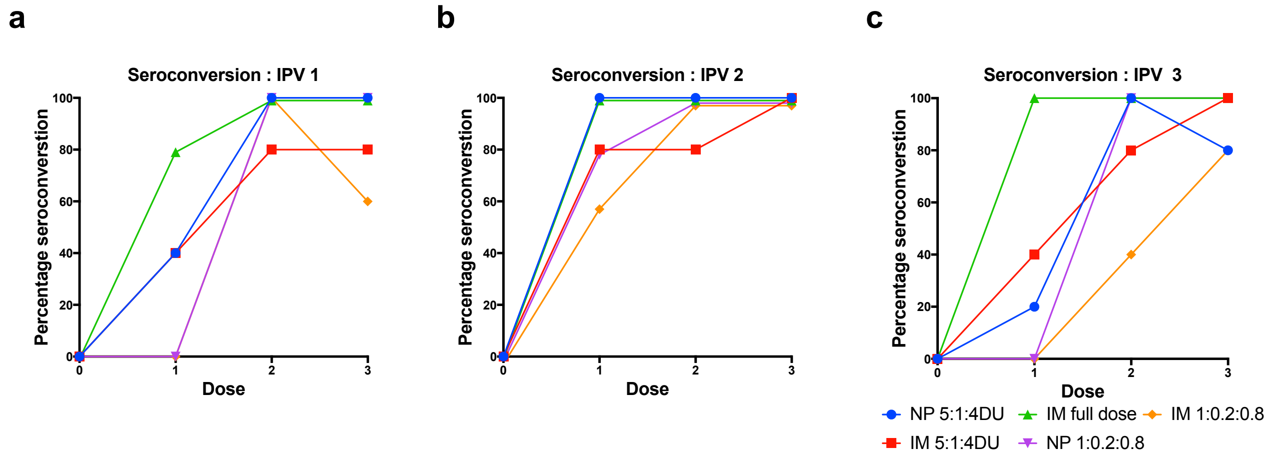

Figure S2. Seroconversion of each group in the triple monovalent study to (**a**) IPV1, (**b**) IPV2 and (**c**) IPV3. Seroconversion percentage corresponds with data presented in **Figure 2a-c**.

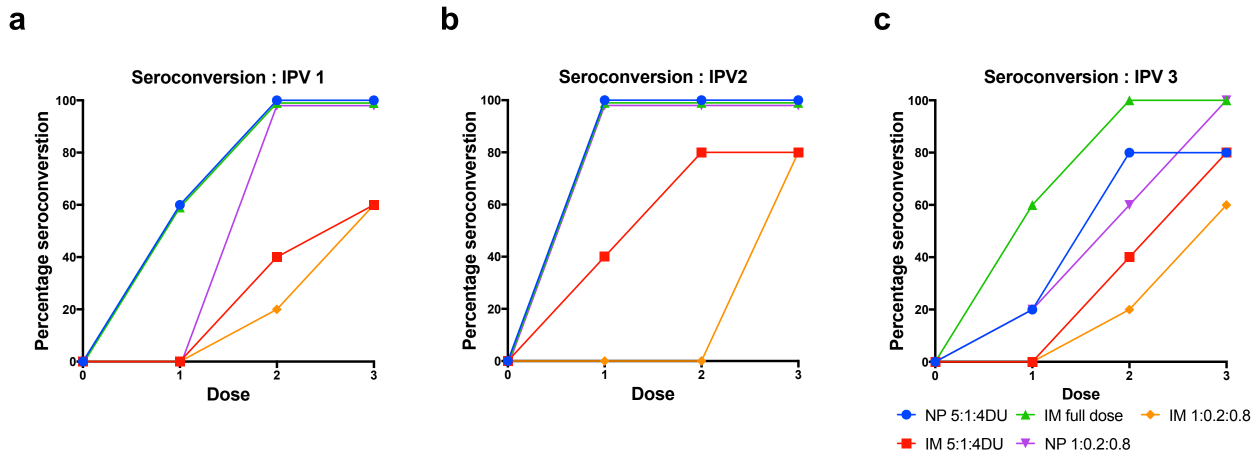

Figure S3. Seroconversion of each group in the trivalent study to (**a**) IPV1, (**b**) IPV2 and (**c**) IPV3. Seroconversion percentage corresponds with data presented in **Figure 4a-c**.
